# The circular RNA circSlc7a11 promotes bone cancer pain pathogenesis in rats by modulating LLC-WRC 256 cell proliferation and apoptosis

**DOI:** 10.1007/s11010-020-04020-1

**Published:** 2021-01-12

**Authors:** Han-Wen Chen, Xiao-Xia Zhang, Zhu-Ding Peng, Zu-Min Xing, Yi-Wen Zhang, Ya-Lan Li

**Affiliations:** 1 Department of Anesthesiology, The First Affiliated Hospital of Jinan University, Tianhe District, No. 613, Huangpu Avenue West, Guangzhou, 510632 China; 2 Department of Anesthesiology, Shunde Hospital, Southern Medical University (The First People’s Hospital of Shunde), Foshan, 528308 China

**Keywords:** circSlc7a11, Bone cancer pain, LLC-WRC 256, Proliferation, Apoptosis

## Abstract

Treatment of bone cancer pain (BCP) caused by bone metastasis in advanced cancers remains a challenge in clinical oncology, and the underlying mechanisms of BCP are poorly understood. This study aimed to investigate the pathogenic roles of circular RNAs (circRNAs) in regulating cancer cell proliferation and BCP development. Eight differentially expressed circRNAs in the rat spinal cord were validated by agarose gel electrophoresis and Sanger sequencing. Expression of circRNAs and mRNAs was detected by quantitative RT-PCR. MTS assay and flow cytometry were performed to analyze cell proliferation and apoptosis, respectively. Differentially expressed mRNA profiles were characterized by deep RNA sequencing, hierarchical clustering, and functional categorization. The interactions among circRNAs, microRNAs (miRNAs), and mRNAs were predicted using TargetScan. Additionally, western blot was performed to determine the protein levels of Pax8, Isg15, and Cxcl10. Multiple circRNAs were differentially expressed in the spinal cords of BCP model rats; of these, circSlc7a11 showed the greatest increase in expression. The overexpression of circSlc7a11 significantly promoted cell proliferation and repressed apoptosis of LLC-WRC 256 and UMR-106 cells, whereas circSlc7a11 silencing produced the opposite effects. Altered expression of circSlc7a11 also induced substantial changes in the mRNA expression profiles of LLC-WRC 256 cells; these changes were linked to multiple apoptotic processes and signaling pathways, such as the chemokine signaling pathway, and formed a complex circRNA/miRNA/mRNA network. Additionally, Pax8, Isg15, and Cxc110 protein level in LLC-WRC 256 cells was consistent with the mRNA results. The circRNA circSlc7a11 regulates rat BCP development by modulating LLC-WRC 256 cell proliferation and apoptosis through multiple-signaling mechanisms.

**Supplementary Information:**

The online version of this article (10.1007/s11010-020-04020-1) contains supplementary material, which is available to authorized users.

## Introduction

Bone cancer pain (BCP) refers to the common, significant, and life-altering pain caused by metastasis into bones that occurs in most patients with cancer at metastatic and advanced stages [1, 2]. As shown by previous clinical research, more than 80% of patients with cancer experience severe BCP as their disease progresses, particularly those with lung, breast, prostate, thyroid, and pancreatic cancers, which usually invade multiple bones, including the spine, ribs, pelvis, femur, tibia, and other long bones [2, 3]. Upon metastasis in bone tissues, cancer cells and neighboring stromal cells secrete various algogenic factors, including bradykinin, protons, endothelins, tyrosine kinase activators, and proteases [1]. Moreover, neuropathic pain can also be caused by damage to the distal ends of nerve fibers because of tumor growth and abnormal formation of sensory and sympathetic nerve fibers, which contribute to the peripheral and central sensitization associated with advance BCP [1, 2]. Although several therapeutic drugs such as opioids, antidepressants, and anticonvulsants can be used to treat BCP, their efficacies in clinics are limited by their severe side effects [2, 3]. Thus, the elucidation of the molecular mechanisms underlying the generation and maintenance of BCP is important for providing new targets for the control and treatment of this condition.

In recent years, BCP development has been shown to be driven by various biological processes and signaling pathways. For example, the activation of spinal C-X-C motif chemokine receptor 3 (CXCR3) promotes central sensitization and BCP generation and maintenance in rat models; this process is mediated by the phosphatidylinositol 3-kinase /protein kinase B pathway as well as the downstream Raf/mitogen-activated protein kinase kinase and extracellular regulated protein kinase signaling cascades [4, 5]. Moreover, many other chemokines, including CXCL12 and its receptor CXCR4, also mediate BCP development, which suggests that they are targets for BCP-controlling drug development [6]. The BCP-promoting role of CXCL12/CXCR4 signaling has mainly been attributed to the sensitization of neurons and the activation of microglia and astrocytes via the c-Jun amino-terminal kinase (JNK) signaling pathway [7]. The activation of glia and the resultant secretion of proinflammatory cytokines, including tumor necrosis factor α (TNF-α), during BCP generation have also been shown to be highly regulated by other signaling pathways, such as the nuclear factor-kappa B (NF-κB) signaling pathway that controls the expression of various pain-related genes [8, 9]. Furthermore, Wnt/β-catenin-signaling pathway is also activated in sensory neurons and astrocytes, and it plays a critical role in neuropathic pain caused by nerve injury and BCP [10]. These collective findings demonstrate the complex signaling events that underlie BCP pathogenesis; however, their regulatory mechanism remains poorly understood.

Circular RNAs (circRNAs) are a large class of non-coding, covalently closed loop-shaped RNAs, which are synthesized via the back splicing of pre-mRNA transcripts [11, 12]. Extensive research in recent decades has disclosed the essential roles played by circRNAs in various biological processes and diseases, including cancer development and metastasis, which are owing to their potent regulation of gene expression through sponging micro RNAs (miRNAs), regulation of transcription, and association with RNA-binding proteins [13–15]. For instance, circ-AKT3, the circular RNA encoded by the protein kinase B gene, has been shown to repress the metastasis of clear cell renal cell carcinoma by promoting E-cadherin expression through sponging miR-296-3p [16]. In breast cancer, circ-DNMT1, which is formed by the back splicing of the DNA methyltransferase 1 transcript, contributes to breast cancer cell proliferation and progression via the activation of cellular autophagy machinery [17]. Moreover, the circRNA hsa_circ_0001564 has been reported to modulate the proliferation and apoptosis of osteosarcoma cells through sponging miR-29c-3p [18]. However, little is known about the pathogenic roles of circRNAs in BCP generation and maintenance. Nevertheless, BCP pathogenesis could be modulated by miR-124 through its regulation of synaptopodin (synpo) protein expression [19], which suggests that circRNAs are involved in BCP as miRNA sponges.

In the present study, based on the sequencing analysis of circRNAs from a previous study, we explored the role of circRNAs in the spinal cord of rat BCP model. We aimed to first characterize the circRNAs that showed significant expressional alterations in rat BCP models and then conduct functional investigations in LLC-WRC 256 cells and identify potential target genes using RNA sequencing. Our analyses provide novel insights into the molecular mechanisms that underlie BCP development, and they might ultimately facilitate the development of new drugs for pain control in patients with metastatic cancer.

## Material and methods

### Cell culture

The rat mammary gland carcinoma cell line LLC-WRC 256 was purchased from the ATCC (American Type Culture Collection) and cultured in RPMI 1640 medium (Thermo Fisher Scientific) supplemented with 10% fetal bovine serum (Gibco) and 1% penicillin–streptomycin (Sigma-Aldrich) at 37 °C in a humidified atmosphere with 5% CO_2_. The rat osteosarcoma cell line UMR-106 was purchased from Guangzhou Cellcook Biotech Co., Ltd. (Guangzhou, China) and maintained in Dulbecco’s-modified Eagle’s medium (Gibco) supplemented with 10% fetal bovine serum (Gibco) at 37 °C in a humidified atmosphere with 5% CO_2_.

### Circular RNA characterization

In our previous study, we successfully created BCP rat models [20]. In this study, the existence of circRNAs in the bone marrow tissues of rat BCP models was characterized by both Sanger sequencing and RT-PCR combined with agarose gel electrophoresis. Briefly, total RNA samples were isolated from rat spinal cords using Trizol solution (#15596026; Thermo Fishier Scientific) following the manufacturer’s suggested protocols. Approximately, 2.5 μg of the RNA samples was used for cDNA synthesis with the Omniscript RT Kit (#205111; Qiagen) following the manufacturer’s instructions. Subsequently, the identities of circRNAs were validated by PCR reactions using the HotStar HiFidelity Polymerase Kit (#202602; Qiagen) and pairs of both convergent and divergent primers amplifying linear RNAs or circRNAs, respectively. Convergent primers were all designed within the same exon. Both cDNA and genomic DNA (gDNA) were applied as the template for PCR analysis. The expression of GAPDH was also analyzed as the control. Moreover, cDNA samples were analyzed by Sanger sequencing to confirm circRNA identities and identify back-splicing junctions.

### Quantitative RT-PCR

Quantitative RT-PCR (qRT-PCR) was performed to analyze the relative expression of mRNAs and circRNAs. Total RNA extraction and cDNA synthesis via reverse transcription were performed as described in the “Circular RNA characterization” subsection. Subsequently, the relative levels of mRNAs or circRNAs were measured using a qRT-PCR assay in which the SYBR™ Select Master Mix (#4472903; Thermo Fishier Scientific) was used according to the manufacturer’s protocol. The expression of GAPDH was also analyzed as the internal standard. The 2^−△△CT^ method was used for final quantitation based on at least three biological repeats. Primers used for quantitation are presented in Table 1.

**Table 1:** Primers for quantitative PCR assay

Primer ID	Primer sequences (5′-3′)	Product length (bp)
R-chr11:34495233|34500943-CF1	TTACTACTGGCTGCCGAGGT	154
R-chr11:34495233|34500943-CR1	TATCCATCTGGAGCCTGTCC	
R-chr11:34495233|34500943-LF1	CGTCTGCAGAGAACATTCCA	248
R-chr11:34495233|34500943-LR1	AGCACTGTCTTCCAGCAGGT	
R-chr17:43258446|43290432-CF1	GGTGAAACGGGCCATACTTA	169
R-chr17:43258446|43290432-CR1	CACTTTGGTCAGGGAGGTGT	
R-chr17:43258446|43290432-LF1	TTTTGGGATGCTTCCTTTTC	167
R-chr17:43258446|43290432-LFR1	GGAGGTCTTCAGCCTGACAT	
R-chr2:139502624|139520450-CF1	GCTGGCTGGTTTTACCTCAA	168
R-chr2:139502624|139520450-CR1	GATGCCCACAGCTGTTACAA	
R-chr2:139502624|139520450-LF1	TTGGGGCTTGGTGTAATCTC	152
R-chr2:139502624|139520450-LR1	GGCCAATGTCTACCAGCAGT	
R-chr3:160491308|160495240-CF1	TGAGGAAGAGGAAGGAACCA	153
R-chr3:160491308|160495240-CR1	CCATTTCTATGGCCGTGATT	
R-chr3:160491308|160495240-LF1	AATGGACTCTGGCACGATGG	172
R-chr3:160491308|160495240-LR1	CCTTCCTCTTCCTCATCCTCC	
R-chr11:47165426|47169764-CF1	GACAGATGAAGTGCGGAGGT	217
R-chr11:47165426|47169764-CR1	GGTGAAGGTCATTCCGAGAG	
R-chr11:47165426|47169764-LF1	TAACTTGGGCCTTGAGGATG	182
R-chr11:47165426|47169764-LR1	TGATCGATGACTTGGGACAA	
R-chr12:8189807|8220963-CF1	CGACTGAGGGACAGATGTGA	249
R-chr12:8189807|8220963-CR1	GTAAACTCCTGCGTGGTGGT	
R-chr12:8189807|8220963-LF1	TGGATACGGTTCTCGGTTTT	154
R-chr12:8189807|8220963-LR1	GCGTACGATCACAGACATGG	
R-chr8:23048110|23048640-CF1	CGTCATGTTCTGGGGTAGGT	187
R-chr8:23048110|23048640-CR1	CAACACTCTCAATGGCCTCA	
R-chr8:23048110|23048640-LF1	CCACCAAGGTCACACTTCCT	157
R-chr8:23048110|23048640-LR1	ACCTACCCCAGAACATGACG	
R-chr9:1442054|1485363-CF1	CTTTGCTCGACCACAGGATT	162
R-chr9:1442054|1485363-CR1	AGGTCGTCCCACTCTCTCAA	
R-chr9:1442054|1485363-LF1	CCATTCCCTTTGCTCGAC	151
R-chr9:1442054|1485363-LR1	AACCCATATATTTGTGGTGGAA	
R-Nptxr-F	GGCCAATGAGATCGTGTTGC	124
R-Nptxr-R	CCAGGCGATGCAGATATGGT	
R-Wnt7b-F	CGCGAGAAGCAAGGCTACTA	194
R-Wnt7b-R	TCCAGTTTCATGCGGTCCTC	
R-Ifit3-F	TCGTCTGAGTGCCCACTTTC	106
R-Ifit3-R	TTGACCTCACTCATGACGGC	
R-Rsad2-F	TTCACGCGTCAGTGCAACTA	188
R-Rsad2-R	CTCACGAGCTTGCCCAAGTA	
R-Cxcl10-F	TGCAAGTCTATCCTGTCCGC	192
R-Cxcl10-R	CTCTCTGCTGTCCATCGGTC	
R-Isg15-F	GAAGGCCCATGGAGGACAAA	128
R-Isg15-R	CATTGGCTCTGGATAGGGGC	
R-Irg1-F	GGAACATTGCAAATGTGTGGGT	151
R-Irg1-R	GTGAACGCTTGCAAGGCAATA	
R-Pax8-F2	CCCACTGCCCTTACTCAACA	143
R-Pax8-R2	CCTGCTTTATGGCGTAGGGT	
R-Rtp3-F2	TGCACCAACTTTGATTTCTGAAG	143
R-Rtp3-R2	TGTGAAGGTCCATTTGTGCC	
R-Oasl-F2	GCAAGGCTACAGGTGGGATA	157
R-Oasl-R2	TAAATCCGGGTGACCCCACT	

### Cell transfection

The sequences of circSlc7a11 were ligated with the pcDNA3.1 vector using NheI (GCTAGC) and EcoRI (GAATTC) as enzymes (Invitrogen). The siRNA sequences targeting circSlc7a11, including siSlc7a11-1 (5′-CAACCCTGAAAACCCCGGA-3′), siSlc7a11-2 (5′-GAAAACCCCGGAGCTACGGCT-3′), and a siRNA negative control (siRNA-NC; 5′-UUCUCCGAACGUGUCACGUTT-3′), were synthesized by the GenePharma Biotech Company (Shanghai, China). Recombinant vectors and siRNAs were introduced into LLC-WRC 256 and UMR-106 cells using Lipofectamine TM 3000 transfection reagent (Thermo Fishier Scientific). Transfection efficacy was evaluated using the qRT-PCR method 48 h later.

### Cell proliferation and apoptosis

Proliferation rates of LLC-WRC 256 and UMR-106 cells were detected using the MTS Cell proliferation kit (#K300-250; BioVision, USA) following the manufacturer’s protocol. Briefly, LLC-WRC 256 and UMR-106 cells cultured in 96-well plates were incubated with 20 µL of MTS solution for 50 min at 37 °C; a microplate reader was then used to detect the OD490 values. The apoptosis of LLC-WRC 256 and UMR-106 cells was analyzed by flow cytometry using the Dead Cell Apoptosis Kit (Annexin V/ADD) (Thermo Fishier Scientific) following the manufacturer’s instructions. Briefly, the cultured LLC-WRC 256 and UMR-106 cells were collected by centrifugation at 600×*g* for 5 min, incubated with Annexin V-FITC and 7-ADD solution for 12 min at room temperature in darkness, and finally measured using flow cytometry.

### Deep sequencing

Differentially expressed mRNAs in LLC-WRC 256 cells were characterized by deep RNA sequencing. Briefly, total RNA samples were isolated from cultured LLC-WRC 256 cells, as described in the “Circular RNA characterization” subsection. The quality of RNA samples was first validated using the Agilent 2100 Bioanalyzer. Subsequently, rRNA molecules were removed using the Qiagen RiboMinus Eukaryote Kit according to the manufacturer’s protocol, and we used poly A enriched RNA. Then, an RNA-seq library was established using the Ultra™ II RNA Library Prep Kit (#E7770S; NEB) following the manufacturer’s instructions. A Hiseq 2000 system (Illumina, USA) was then used to sequence the RNA samples, and clean reads from sequencing were aligned with the rat reference genome database using Bowtie 2 (Version: 2.3.5.1) [21]. The relative expression of mRNAs between groups was quantitated using TPM values. A false discovery rate of < 0.01 and a log_2_Ratio of > 1 was applied together as the threshold for significantly differential expression.

### Bioinformatics

The hierarchical clustering of differentially expressed mRNAs in LLC-WRC 256 cells was completed using the R software package (Version 1.0.8). Functional categorization based on Gene Ontology (GO) biological processes and signaling pathways was conducted using the Database for Annotation, Visualization and Integrated Discovery and the Kyoto Encyclopedia of Genes and Genomes database, respectively. The interaction network incorporating circRNAs, miRNAs, and mRNAs was predicted using TargetScan [22].

### Western blot

Total protein was extracted from LLC-WRC 256 cells, loaded on SDS-PAGE, and transferred to PVDF membranes. Nonfat milk in PBS was used to block the membrane at room temperature for 1 h. The membrane was incubated overnight at 4 °C with primary antibodies against Pax8 (#ab97477, Abcam), Isg15 (#ab227541, Abcam), Cxcl10 (#ab271239, Abcam) and GAPDH (#ab181602, Abcam). Next, the membrane was washed with TBST three times and incubated with a corresponding secondary antibody at 37 °C for 45 min. Target bands were visualized using enhanced chemi-luminescence (Bio-Rad). GAPDH served as an internal control.

### Statistical analysis

SPSS 20.0 was used to compare the quantitative data in the present study. Differences between groups were analyzed using Student’s *t* test and ANOVA. *P* values of < 0.05 were considered statistically significant.

## Results

### Differentially expressed circRNAs in the spinal cord of BCP model rats

**Fig. 1:**
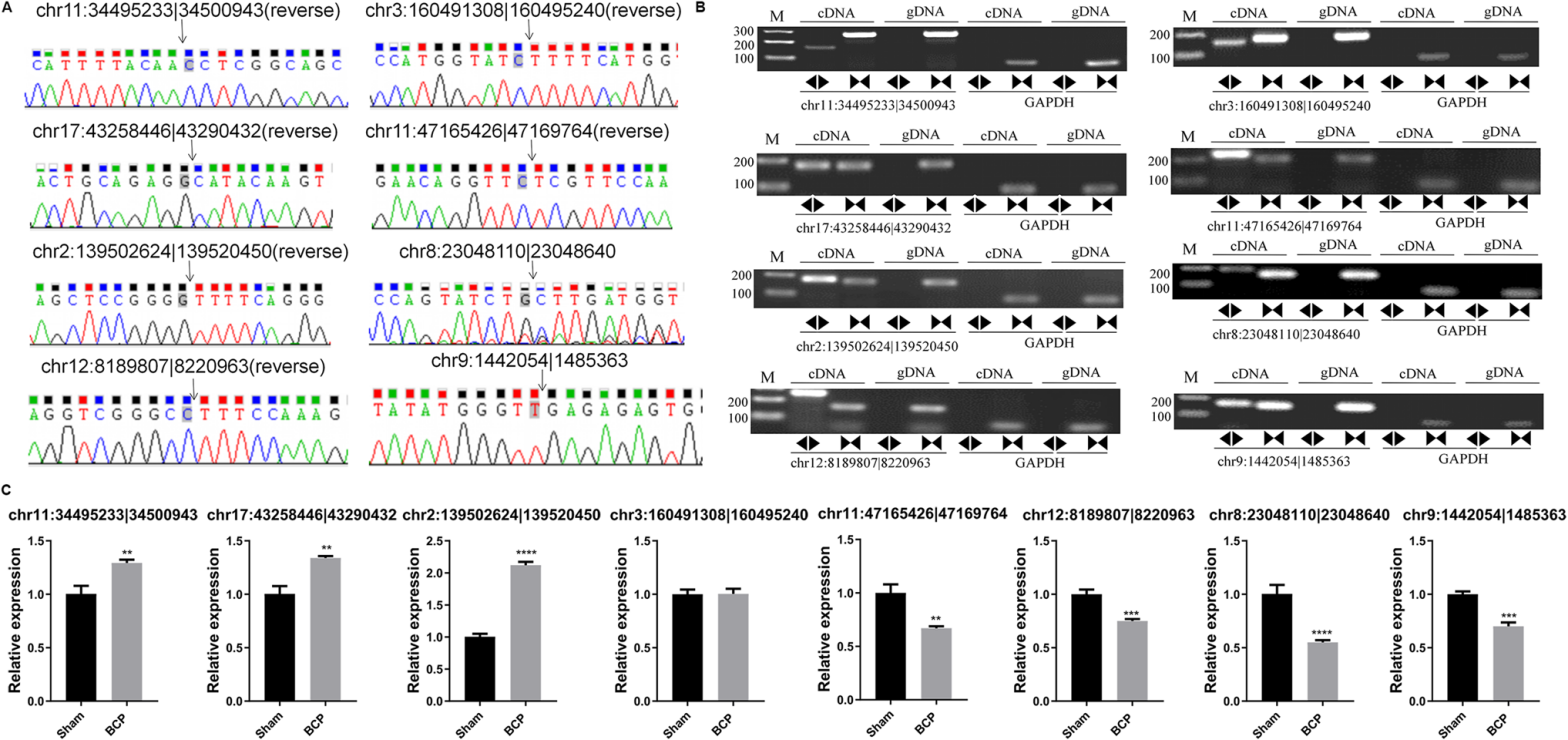
Significant alterations in circRNA expression in the spinal cords of BCP model rats. **a** Validation of circRNA expression and back-splicing junction characterization in BCP model rats. The back-splicing junctions of circRNAs identified by Sanger sequencing are indicated by black arrows. **b** Confirmation of the circular morphology of circRNAs through RT-PCR combined with agarose gel electrophoresis. The circular and linear RNAs were detected using divergent and convergent primers, respectively, from cDNA templates. Genomic DNA and primers targeting GAPDH were used as controls. **c** Differential expression of circRNAs in the spinal cords of Sham and BCP model rats. Quantitative RT-PCR was used to analyze circRNA expression. gDNA: genomic DNA; GAPDH: glyceraldehyde-3-phosphate dehydrogenase; BCP: bone cancer pain. **P < 0.01; ***P < 0.001; ****P < 0.0001

To investigate the potential involvement of circRNAs in BCP development, eight circRNAs were selected for validation. Their selection was based on the length of circRNA (500–2000 bp), differential expression relative to the control (being in the “top 15” upregulated or downregulated circRNAs), and their regulation of miRNAs (related to BCP, breast cancer osteosarcoma, and lung cancer) (Supplemental Table 1). Through Sanger DNA sequencing, we also identified their back-splicing junctions (Fig. 1a). Moreover, the circular structures of these eight circRNAs were validated through RT-PCR combined with agarose gel experiment using divergent primers and a cDNA template, which were not amplified from gDNA templates (Fig. 1b). Furthermore, no signals were observed during RT-PCR assays with divergent primers targeting GAPDH, which served as a negative control (Fig. 1b). Therefore, these analyses verified the expression of the eight circRNA candidates in the spinal cords of BCP model rats. Consequently, we detected the expressional alterations of the circRNAs between the Sham and BCP model groups using qRT-PCR (Fig. 1c). We found that the expression of three circRNAs significantly increased in the BCP group relative to the Sham group, whereas that of four circRNAs significantly decreased (Fig. 1c). Among these, circSlc7a11 (chr2:139502624|139520450), which was formed through back splicing of the pre-mRNA of the cystine–glutamate transporter solute carrier family 7 member 11 cystine–glutamate transporter (*Slc7a11*), exhibited the most significant expressional increase in the BCP group (Fig. 1c); hence, circSlc7a11 was selected for further analyses.

### circSlc7a11 promotes proliferation and represses apoptosis of LLC-WRC 256 and UMR-106 cells

**Fig. 2:**
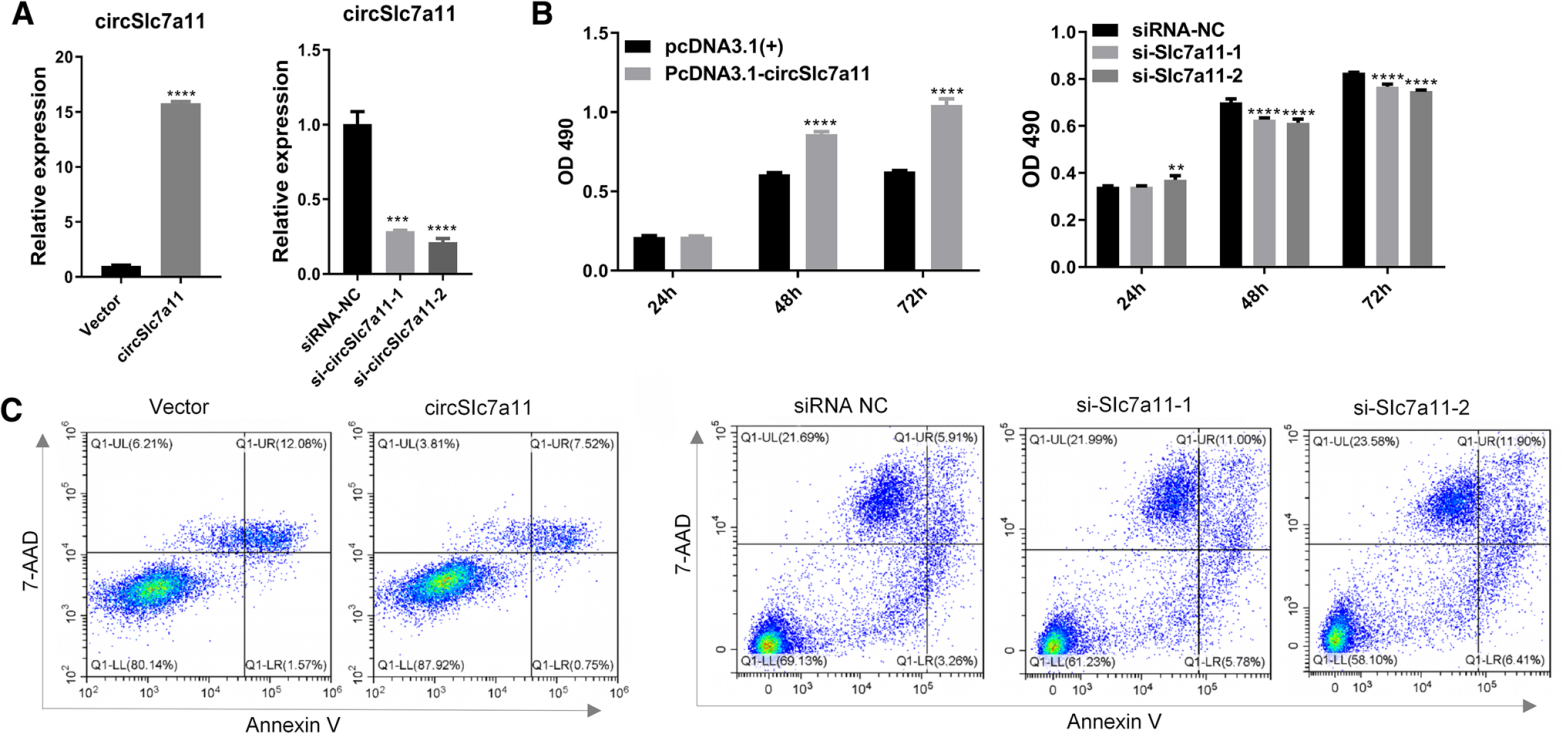
Regulation of LLC-WRC 256 cell proliferation and apoptosis by circSlc7a11. **a** Alterations of circSlc7a11 expression in LLC-WRC 256 cells by transfection with overexpressing vectors or siRNAs. The expression of circSlc7a11 in cells was detected using quantitative RT-PCR. **b** Positive regulation of LLC-WRC 256 cell proliferation by circSlc7a11. The proliferation rates of LLC-WRC 256 cells with altered circSlc7a11 expression were measured using an MTS assay. **c** Inhibition of LLC-WRC 256 cell apoptosis by circSlc7a11. The percentage of apoptotic LLC-WRC 256 cells was determined by flow cytometry. *Slc7a11*: solute carrier family 7 member 11 cystine–glutamate transporter; NC: negative control; OD490: absorbance at 490 nm; **P < 0.01; ***P < 0.001; ***P < 0.0001

**Fig. 3:**
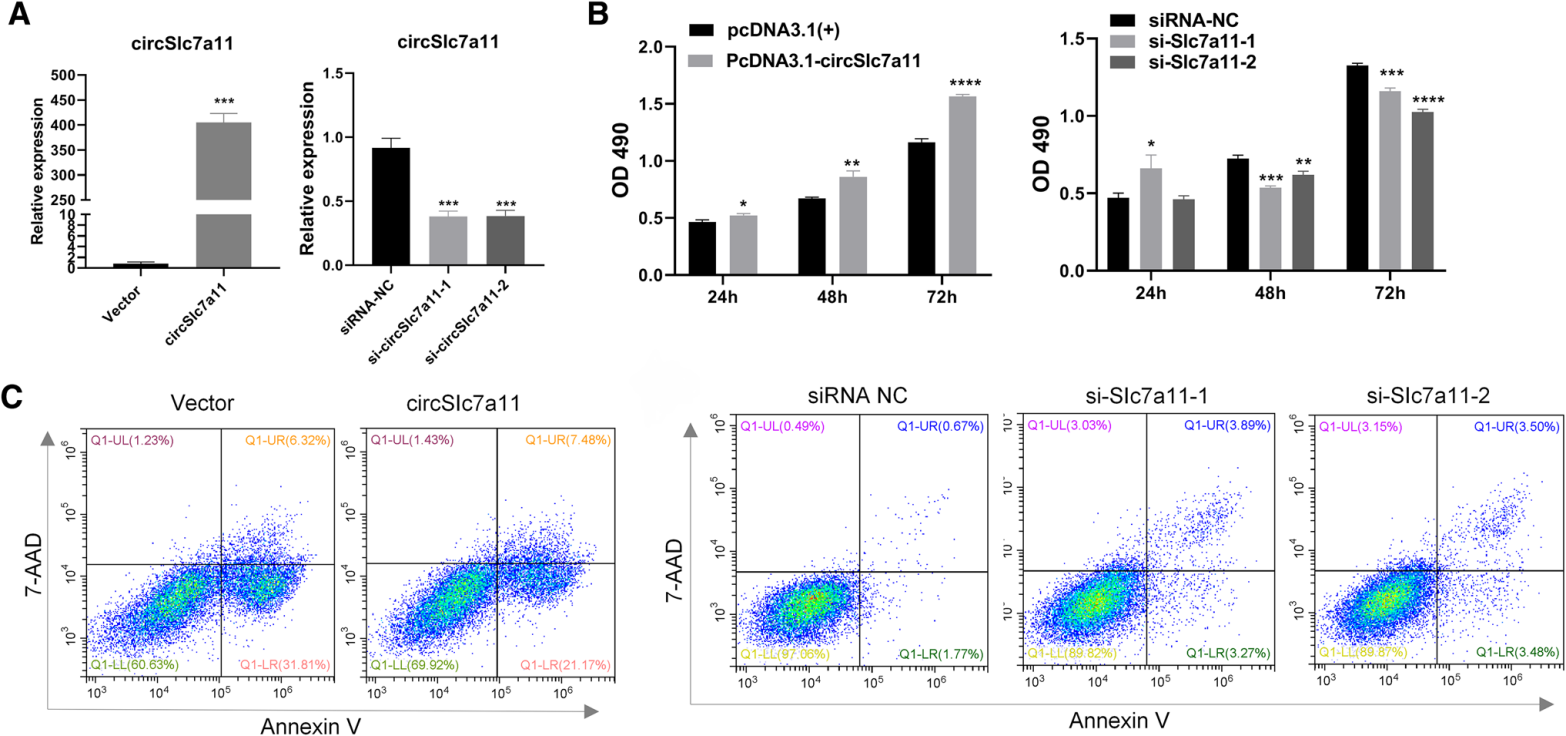
Regulation of UMR-106 cell proliferation and apoptosis by circSlc7a11. **a** Alterations of circSlc7a11 expression in UMR-106 cells by transfection with overexpressing vectors or siRNAs. The expression of circSlc7a11 in cells was detected using quantitative RT-PCR. **b** Positive regulation of UMR-106 cell proliferation by circSlc7a11. The proliferation rates of UMR-106 cells with altered circSlc7a11 expression were measured using MTS assay. **c** Inhibition of UMR-106 cell apoptosis by circSlc7a11. The percentage of apoptotic UMR-106 cells was determined by flow cytometry. *Slc7a11*: solute carrier family 7 member 11 cystine–glutamate transporter; NC: negative control; OD490: absorbance at 490 nm; *P < 0.05; **P < 0.01; ***P < 0.001; ***P < 0.0001

To analyze the cellular function of circSlc7a11, we modulated the expression of circSlc7a11 in LLC-WRC 256 and UMR-106 cells. Transfection with overexpression vectors or siRNAs resulted in the significant elevation or suppression of circSlc7a11 in LLC-WRC 256 cells relative to the control group, indicating that the overexpression vectors and siRNAs had been successfully transfected into LLC-WRC 256 cells (Fig. 2a). In LLC-WRC 256 cells with overexpression of circSlc7a11, we found that proliferation rates were remarkably enhanced compared with rates in cells transfected with the empty vectors (Fig. 2b). In contrast, si-circSlc7a11 transfection greatly repressed the proliferation of LLC-WRC 256 cells relative to that of the negative control cells (Fig. 2b). Furthermore, flow cytometry showed that the apoptosis of LLC-WRC 256 cells was substantially repressed by overexpression of circSlc7a11 but markedly promoted by the silencing of circSlc7a11 with siRNAs (Fig. 2c). As shown in Fig. 3a–c, the expression of circSlc7a11 in UMR-106 cells was consistent with that in LLC-WRC 256 cells. Thus, these results indicate that circSlc7a11 possesses potent proliferation-promoting and apoptosis-inhibiting abilities in carcinoma cells.

### Differential gene expression profiles induced by circSlc7a11 in LLC-WRC 256 cells

**Fig. 4:**
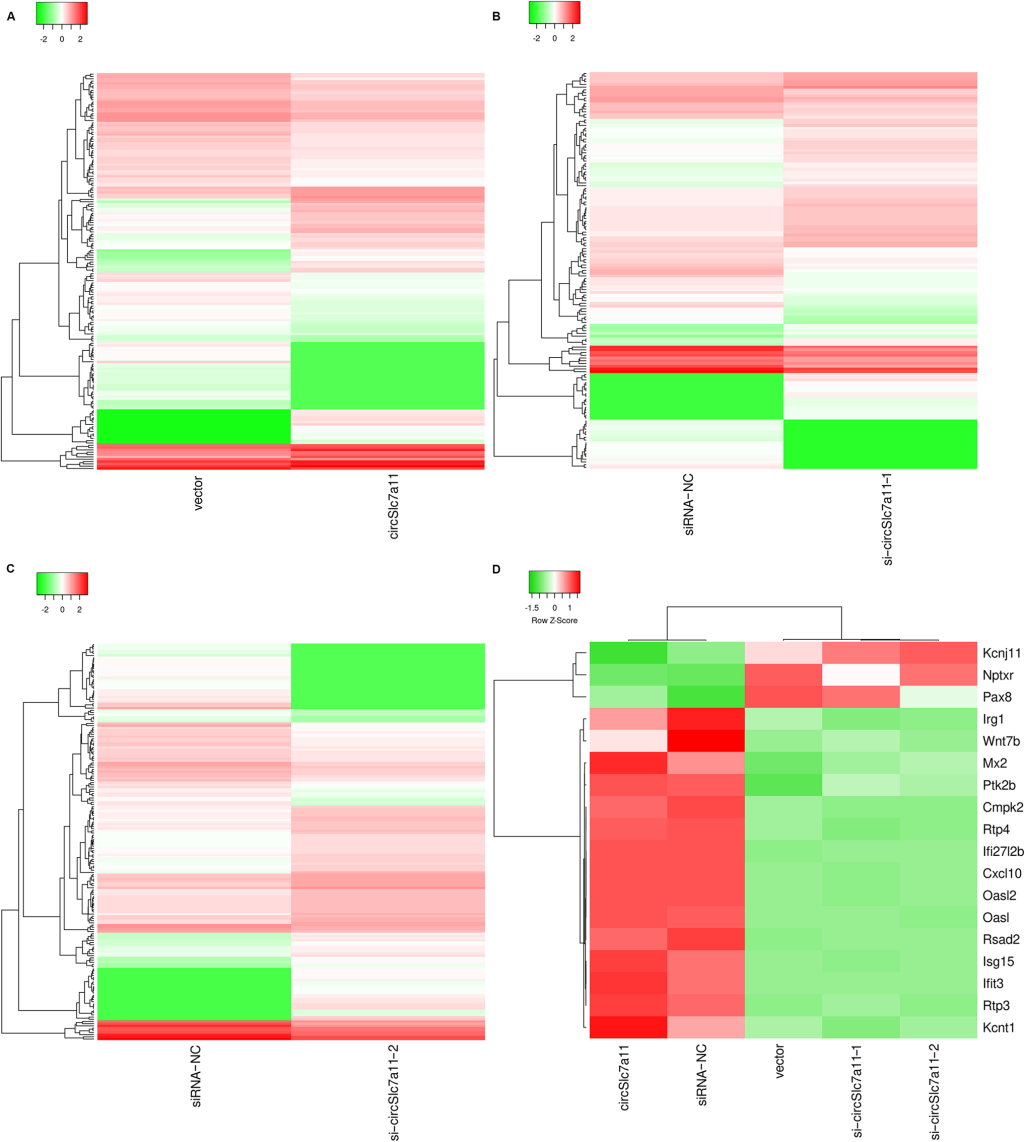
Significant mRNA profile changes induced by circSlc7a11 in LLC-WRC 256 cells. Differentially expressed mRNAs in LLC-WRC 256 cells with altered circSlc7a11 expression were characterized by deep RNA sequencing. Hierarchical clustering was performed to analyze differentially expressed genes in circSlc7a11-overexpressing LLC-WRC 256 cells (**a**), as well as those transfected with si-circSlc7a11-1 (**b**), and si-circSlc7a11-2 (**c**). In total, 18 genes were differentially expressed in LLC-WRC 256 cells by either circSlc7a11 overexpression or silencing (**d**). Upregulated and downregulated expression are presented in red and green, respectively. NC: negative control

To elucidate the molecular mechanisms mediating the regulation of LLC-WRC 256 cells by circSlc7a11, we comprehensively characterized the differentially expressed mRNAs in LLC-WRC 256 cells with altered circSlc7a11 expression using deep RNA sequencing (Fig. 4). In total, 171 mRNAs were differentially expressed between LLC-WRC 256 cells transfected with circSlc7a11-overexpressing vectors and those with empty vectors: 61 genes were upregulated and 110 were downregulated (Fig. 4a; Supplemental Table 2). Moreover, the expression of 145 and 181 mRNAs was significantly altered between LLC-WRC 256 cells transfected with siRNA-NC and si-circSlc7a11-1 (Fig. 4b; Supplemental Table 3) and those transfected with siRNA-NC and si-circSlc7a11-2 (Fig. 4c; Supplemental Table 4), respectively. With further analysis, we found that 18 differentially expressed genes co-existed in the sequencing analysis shown in Figs. 3a–c and 4d. For instance, the expression of the ATP-sensitive potassium channel subunit Kir6.2 (*Kcnj11*), neuronal pentraxin receptor-1 (*Nptxr*), and paired box 8 (*Pax8*) was reduced by circSlc7a11 overexpression and enhanced by circSlc7a11 silencing in LLC-WRC 256 cells (Fig. 4d). Conversely, the expression of immune responsive gene 1 (*Irg1*), wingless/integrated 7b (*Wnt7b*), Myxovirus Resistance 2 (*Mx2*), and 12 other genes in LLC-WRC 256 cells was increased by circSlc7a11 overexpression and repressed by circSlc7a11 silencing (Fig. 4d).

### circSlc7a11 regulates various biological processes and signaling networks in LLC-WRC 256 cells

**Fig. 5:**
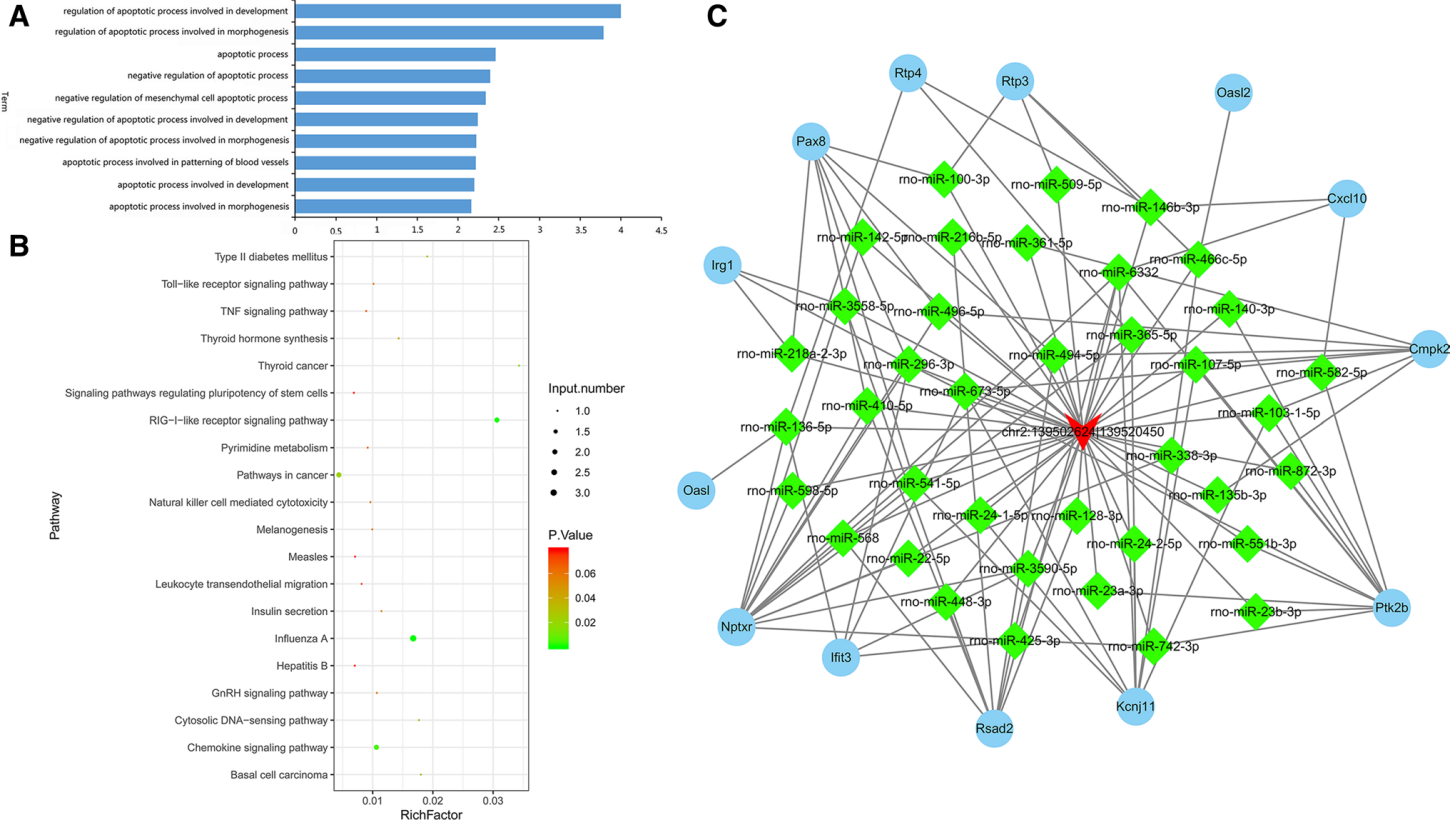
Biological processes and signaling networks regulated by circSlc7a11 in LLC-WRC 256 cells. **a** Functional categorization of the 18 mRNAs differentially expressed in LLC-WRC 256 cells with altered expression of circSlc7a11. GO categorization was performed based on biological processes. **b** The KEGG signaling pathways associated with the 18 differentially expressed mRNAs. **c** The circRNA-miRNA-mRNA interaction network regulated by circSlc7a11. The interactions between circSlc7a11 and nine differentially expressed mRNAs and potential miRNAs were predicted using TargetScan

To further explore the mechanisms underlying circSlc7a11-regulated LLC-WRC 256 cell functions, we performed bioinformatics analyses of the biological processes and signaling pathways associated with the 18 differentially expressed mRNAs induced by circSlc7a11. Based on GO biological processes, these differentially expressed mRNAs were significantly enriched in multiple apoptotic processes linked to development, morphogenesis, mesenchymal cells, and patterning of blood vessels (Fig. 5a). These circSlc7a11-regulated mRNAs were also involved in many signaling pathways, including RIG-I-like receptor signaling, chemokine signaling, Toll-like receptor signaling, TNF signaling, GnRH signaling, as well as pathways involved in cancer, influenza A, and pyrimidine metabolism (Fig. 5b).

**Fig. 6:**
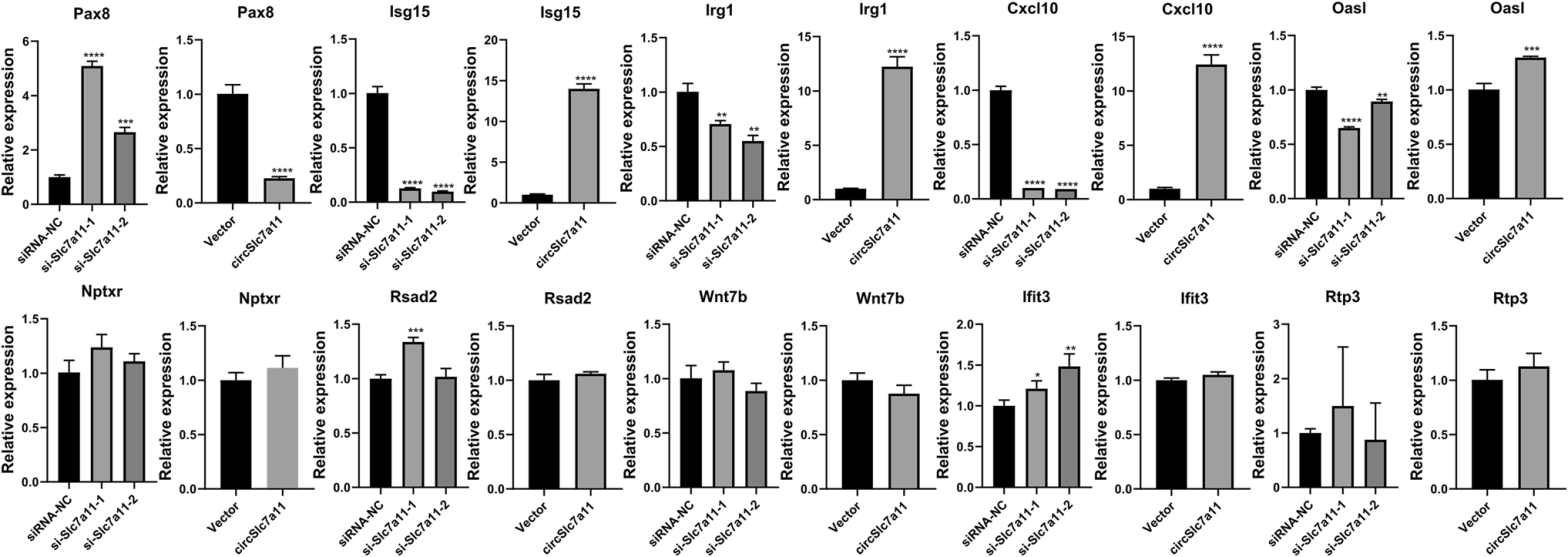
Validation of 10 differentially expressed mRNAs in LLC-WRC 256 cells with elevated or silenced circSlc7a11. The expression of mRNAs was determined by quantitative RT-PCR. *Pax8*: paired box 8; *Isg15*: interferon-stimulated gene 15; *Irg1*: immune responsive gene 1; *Cxcl10*: C-X-C motif chemokine ligand 10; *Oasl*: Oligoadenylate Synthetase-like; *Nptxr*: neuronal pentraxin receptor-1; *Rsad2*: Radical S-adenosyl methionine domain containing 2; *Wnt7b*: wingless/integrated 7b; *Ifit3*: Interferon-Induced Protein with Tetratricopeptide Repeats 3; *Rtp3*: receptor transporting protein 3. *P < 0.05; **P < 0.01; ***P < 0.001; ***P < 0.0001

**Fig. 7:**
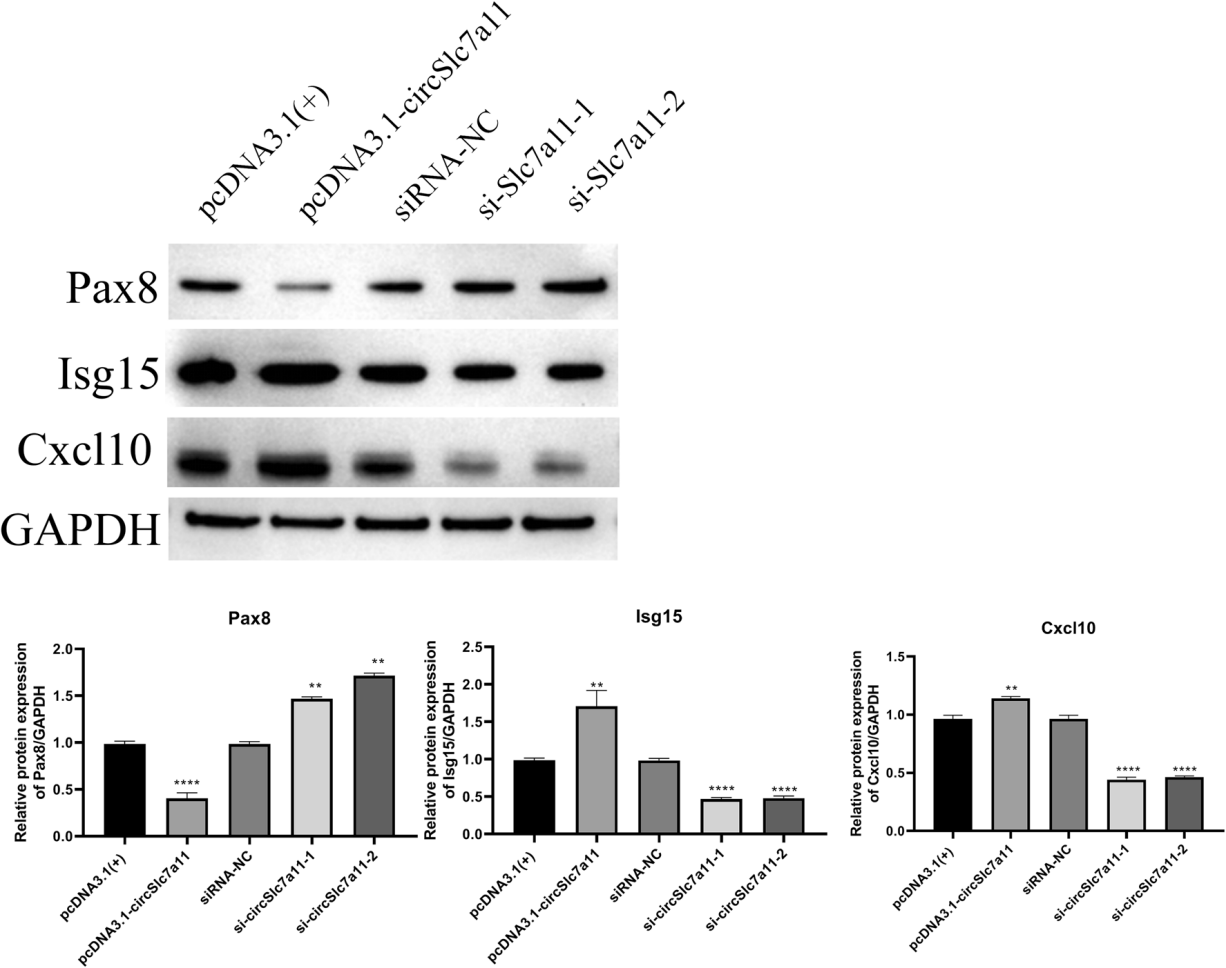
Western blot was performed to evaluate Pax8, Isg15, and Cxcl10 expression levels in LLC-WRC 256 cells. The relative expression levels of all proteins were calculated based on the gray values in each group. *n* = 3; **P < 0.01; ****P < 0.0001. Gray values indicate relative expression levels of proteins

Using TargetScan for predictions, we showed that circSlc7a11 might also regulate the expression of these mRNAs via complex interactions with a number of miRNAs (Fig. 5c; Supplemental Table 5). For instance, the expression of *Kcnj11* was predicted to be modulated by circSlc7a11 through interactions with miR-140-3p, miR-673-5p, miR-425-3p, and miR-365-5p (Fig. 5c). Finally, the expression of 10 mRNAs in LLC-WRC 256 cells in which circSlc7a11 was overexpressed or silenced was confirmed using qRT-PCR; these included *Pax8*, interferon-stimulated gene 15 (*Isg15*), Irg1, C-X-C motif chemokine ligand 10 (*Cxcl10*), oligoadenylate synthetase-like (*Oasl*), *Nptxr*, radical S-adenosyl methionine domain containing 2 (*Rsad2*), *Wnt7b*, interferon-induced protein with tetratricopeptide repeats 3 (*Ifit3*), and receptor transporting protein 3 (*Rtp3*) (Fig. 6). Among these, the expression of *Pax8*, *Isg15*, *Irg1*, *Cxcl10*, and *Oasl* was consistent with that in the RNA-seq results. Next, we detected the protein levels of Pax8, Isg15, and Cxcl10 using western blotting. With the overexpression of circSlc7a11, Pax8 protein level was downregulated and Isg15 and Cxcl10 protein levels were upregulated. With the silencing of circSlc7a11, the Pax8 expression was upregulated and the Isg15 and Cxcl10 expressions were downregulated (Fig. 7). Of these, the protein levels of Pax8, Isg15, and Cxcl10 in LLC-WRC 256 cells was consistent with the mRNA results. Cumulatively, these results indicate that the regulation of LLC-WRC 256 cell function and BCP by circSlc7a11 was mediated by various signaling pathways involving multiple miRNAs and functional genes.

## Discussion

Considering the constantly high incidences of cancer recorded over recent decades, BCP caused by bone metastasis of multiple cancers at advanced stages remains a challenging medical problem worldwide [2, 3]. Although the generation and progression of BCP have previously reported to be mediated by various signaling pathways [4, 5], the epigenetic mechanisms underlying BCP pathogenesis mediated by non-coding RNAs have not been studied in detail. Therefore, in the present study, we focused on the expressional alterations and pathogenic roles of circRNAs in a rat BCP model established through intratibial inoculation of rat LLC-WRC 256 cells in tibia tissues. The existence and expressional alteration of multiple circRNA candidates was validated in this rat BCP model; however, expression of the circRNA circSlc7a11 formed via *Slc7a11* pre-mRNA back splicing was found to be substantially elevated in BCP rats. We confirmed that overexpression or silencing of circSlc7a11 substantially altered the proliferation and apoptosis of LLC-WRC 256 and UMR-106 cells. The altered mRNA profiles of LLC-WRC 256 cells with elevated or silenced circSlc7a11 expression were also characterized; changes involved multiple biological processes and signaling pathways mediated by a complex circRNA/miRNA/mRNA interaction network. Taken together, these observations reveal the novel epigenetic mechanisms underlying BCP development mediated by circRNA-regulated gene expression and signaling cascades.

In past decades, the roles of non-coding RNAs in cancer cells have been extensively studied mainly because of their potent epigenetic regulation of functional gene expression and signaling transduction [23–25]. The circRNAs, generated through back splicing of pre-mRNAs, have also been characterized as essential regulators of cancer initiation and progression, as miRNA sponges, and as transcription modulators [13, 15]. Although circRNAs have been associated with many aspects of cancer pathogenesis, little is currently known about their involvement in the generation and maintenance of severe pain caused by bone metastasis. A previous report showed that the neuropathic pain induced by nervous system damage in the spared nerve injury model was accompanied by significant changes in non-coding RNA expression, including the expression of long non-coding RNAs (lncRNAs), miRNAs, and circRNAs [26]. Moreover, the circRNA circAnks1a in dorsal horn neurons was shown to modulate hypersensitivity and neuropathic pain associated with nerve injury through sponging miR-324-3p to regulate the expression of the vascular endothelial growth factor β [27]. In the present study, we observed changes in the expression of multiple circRNAs, with a focus on circSlc7a11, in the rat BCP model, which provides further evidence to support the hypothesis that circRNAs function in the generation of pain in cancer metastasis and other pathogenic conditions.

The pathogenesis of BCP has been attributed to the high proliferation and metastatic capacities of malignant cancer cells, which cause severe damage to bone structure, including fractures [1]. In the present study, we showed that the overexpression of circSlc7a11 significantly promotes the proliferation of LLC-WRC 256 and UMR-106 cells and inhibits apoptosis, whereas circSlc7a11 silencing exerts the opposite effects; these findings indicate that the functions of circSlc7a11 in BCP pathogenesis are at least partially owing to the regulation of cancer cell proliferation and invasion by circRNA. circSlc7a11 is formed by pre-mRNA back splicing of the *Slc7a11*, which encodes a cystine–glutamate transporter protein with various biological and pathogenic functions, including epileptic seizures and osteoporosis [28, 29]. Moreover, *Slc7a11* has been reported to regulate several tumorigenic events, such as breast cancer apoptosis, poor glioblastoma survival, and cancer glucose metabolism and dependence [30–32]. Considering our observation that the proliferation and apoptosis of LLC-WRC 256 and UMR-106 cells were regulated by changes in circSlc7a11 expression, we have now revealed another mechanism of *Slc7a11* functioning in carcinogenesis. Another recent study showed that the antisense lncRNA As-SLC7A11 also regulates ovarian cancer cell migration by targeting *Slc7a11* expression [33]. These findings suggest that *Slc7a11* and related non-coding RNAs have multifaceted roles in cancer development and BCP pathogenesis; therefore, this research area deserves further investigation.

Our deep sequencing analysis revealed that differentially expressed genes in LLC-WRC 256 cells with overexpression or silencing of circSlc7a11 were enriched in many apoptotic processes, which is consistent with our findings of altered apoptosis in treated LLC-WRC 256 cells. More importantly, we also characterized multiple signaling pathways with significant enrichment of these differentially expressed genes. For example, the chemokine signaling pathway has been previously established as a critical regulator of BCP generation and maintenance, and it is regarded as a promising target for BCP control and treatment [5, 6]. Specifically, the chemokine CXCL10 regulates bone pain induced by metastatic breast cancer in rats by activating microglia [34]. In the present study, *Cxcl10* expression in LLC-WRC 256 cells increased and decreased with circSlc7a11 overexpression and repression, respectively, which provides evidence for the mediating role of chemokine signaling in circSlc7a11-regulated BCP development. Moreover, we predicted a complex interaction network regulated by circSlc7a11 and many miRNAs and mRNAs such as *Pax8*, *Isg15*, *Irg1*, *Cxcl10*, and *Oasl*. Western blotting showed that Pax8, Isg15, and Cxcl10 protein levels in LLC-WRC 256 cells were consistent with the mRNA results. Among these, Pax8 immunostaining has previously used for the diagnosis of malignant ovarian carcinoma with chest pain owing to bone metastasis [35], whereas Isg15, an ubiquitin-like protein that functions in autophagy-mediated protein lysosomal degradation, was recently shown to be involved in inflammatory osteolysis through regulation of the NF-kB signaling and bone erosion [36].

However, this study has some limitations. Based on the methods and results we have obtained, this study should be considered a pilot study. But these results might provide some assistance for further studies on the molecular mechanisms underlying BCP. Therefore, we will make further experiments, such as creating a model to demonstrate the effect on biological processes and signaling pathways, studying how these pathways affect apoptosis, and elucidating the pathogenic roles of these genes and interacting miRNAs in BCP development, to study the molecular mechanisms underlying BCP and the comprehensive experiments will be performed and reported in future.

In summary, we have characterized circSlc7a11 as a circRNA that is highly expressed in the rat BCP model and modulates the proliferation and apoptosis of LLC-WRC 256 cells through multiple biological processes and signaling pathways, possibly via its widespread interactions with miRNA and mRNA expression. Therefore, our investigation provides novel insights into the molecular mechanisms underlying BCP pathogenesis, and our findings could serve as the basis for developing new drugs for BCP treatment.

## Supplementary Information

Below is the link to the electronic supplementary material.

(xlsx 11 kb)

(xlsx 27 kb)

(xlsx 24 kb)

(xlsx 27 kb)

(xlsx 12 kb)

## Data Availability

The datasets generated during and/or analysed during the current study are available from the corresponding author on reasonable request.
